# Effectiveness of Digital Cognitive Behavioral Therapy for Insomnia in Young People: Preliminary Findings from Systematic Review and Meta-Analysis

**DOI:** 10.3390/jpm12030481

**Published:** 2022-03-16

**Authors:** Hsin-Jung Tsai, Albert C. Yang, Jun-Ding Zhu, Yu-Yun Hsu, Teh-Fu Hsu, Shih-Jen Tsai

**Affiliations:** 1 Institute of Brain Science, National Yang Ming Chiao Tung University, Taipei 112304, Taiwan; hjtsai15@gmail.com (H.-J.T.); accyang@gmail.com (A.C.Y.); junding0818.y@nycu.edu.tw (J.-D.Z.); 2 Digital Medicine Center, National Yang Ming Chiao Tung University, Taipei 112304, Taiwan; yunhau0808@gmail.com; 3 Department of Emergency Medicine, Taipei Veterans General Hospital, Taipei 11217, Taiwan; tfhsu@vghtpe.gov.tw; 4 Center for Evidence-Based Medicine, Taipei Veterans General Hospital, Taipei 11217, Taiwan; 5 Department of Psychiatry, Taipei Veterans General Hospital, Taipei 11217, Taiwan

**Keywords:** telehealth, digital sleep medicine, student, youth, sleep health, smart healthcare

## Abstract

Various forms of cognitive behavioral therapy for insomnia (CBT-i) have been developed to improve its scalability and accessibility for insomnia management in young people, but the efficacy of digitally-delivered cognitive behavioral therapy for insomnia (dCBT-i) remains uncertain. This study systematically reviewed and evaluated the effectiveness of dCBT-i among young individuals with insomnia. We conducted comprehensive searches using four electronic databases (PubMed, Cochrane Library, PsycINFO, and Embase; until October 2021) and examined eligible records. The search strategy comprised the following three main concepts: (1) participants were adolescents or active college students; (2) dCBT-I was employed; (3) standardized tools were used for outcome measurement. Four randomized controlled trials qualified for meta-analysis. A significant improvement in self-reported sleep quality with a medium-to-large effect size after treatment (Hedges’s *g* = −0.58~−0.80) was noted. However, a limited effect was detected regarding objective sleep quality improvement (total sleep time and sleep efficiency measured using actigraphy). These preliminary findings from the meta-analysis suggest that dCBT-i is a moderately effective treatment in managing insomnia in younger age groups, and CBT-i delivered through the web or a mobile application is an acceptable approach for promoting sleep health in young people.

## 1. Introduction

Insomnia is a particularly prevalent sleep disorder in the general population, including in adolescence and early adulthood [1]. Insomnia disorder is defined by its symptoms (difficulty in initiating sleep, maintaining sleep, or early morning awakening), frequency (at least three times per week and lasting for at least three months), and the significant daytime function impairment it causes, as well as distress to the patient [2]. Notably, insomnia was reported to be prevalent in young students during the coronavirus 2019 (COVID-19) pandemic, with 18%, 25.3%, and 25.7% of 11,835 students from middle school, high school, and university, respectively, experiencing insomnia symptoms in 2020 [3]. Moreover, rates of significant dissatisfaction with sleep quality (SQ), evaluated using the Pittsburgh Sleep Quality Index [4] (PSQI; PSQI ≥ 5), before and during the pandemic were 58% and 73.3% among university students, respectively. Likewise, 16.3% of 307 university students recorded a score higher than 15 on the Insomnia Severity Index (ISI) [5], which is the clinical definition of insomnia, during the COVID-19 pandemic compared with 6.9% before the pandemic [6]. Insomnia and insufficient sleep in adolescents [1] or people in early adulthood are strongly linked to adverse effects on mental health, including depression [7,8], suicidal intention [9], and substance abuse [10]. Contrarily, a protective effect of high-quality sleep on illicit drug abuse was observed [10].

Cognitive behavioral therapy for insomnia (CBT-i) [11] has been strongly recommended by the American College of Physicians [12], American Academy of Sleep Medicine [13], and European Sleep Research Society [14] as the first-line nonpharmacological treatment for chronic insomnia at all ages, as it is advantageous for its low potential for developing tolerance and the long-term durability of treatment gains [15]. CBT-i is a multimodal treatment, with educational (sleep physiology or healthy behaviors), cognitive (restructuring negative sleep beliefs or reducing excessive worry about sleep), and behavioral (sleep restriction and stimulus control therapy) components as well as relaxation techniques (progressive muscle relaxation or imagery relaxation to promote sleep) [16].

Although numerous empirical studies have demonstrated the effectiveness of face-to-face CBT-i in treating insomnia [17] as well as its suitability for individuals with insomnia comorbid with mental illness [18,19], the treatment duration, long waiting time for treatment, travel expenses, and shortage of professional personnel limit its acceptability and scalability [20,21,22]. The COVID-19 pandemic intensified these challenges, leading to a crucial reassessment and restructuring of CBT-I delivery that did not compromise its effectiveness. For instance, delivering CBT-i through digital approaches can eliminate geographic factors, promote sleep health in the general population, and complement the standardized CBT-i procedure performed by sleep specialists [23].

Several considerations have arisen regarding digital cognitive behavioral therapy for insomnia (dCBT-i), such as how to deliver the treatment and the appropriate level of personal support [24]. dCBT-i has grown over the last decade with the development of web- and mobile-based CBT-i. dCBT-i programs are commonly structured with the key components of CBT-i, including sleep education, cognitive therapy, sleep restriction, stimulus control, and relaxation techniques. Moreover, most dCBT-i programs provide degrees of personal support (i.e., sending of regular email reminders and text messages or alerts when users do not log in) to enhance engagement. Users can also evaluate their sleep status using online sleep diaries, sleep questionnaires, or by connecting the program with a wrist-worn actigraph to track daily sleep patterns for objective SQ assessment [25].

In 2016, a meta-analysis of 11 randomized controlled trials (RCTs) concluded that dCBT-i has robust efficacy in the adult population [26]. The trials indicated significantly reduced insomnia severity (*d* = 0.89), decreased self-reported sleep onset latency (SOL; *d* = 0.34), decreased amounts of time spent awake during the night (*d* = 0.45), and enhanced sleep efficiency (SE; *d* = 0.49), as well as satisfaction in SQ (*d* = 0.40) immediately after treatment. These findings were congruent with those of a meta-analysis conducted in 2012 [27] that reported medium (SE, SQ, SOL, and number of awakenings, reported with a sleep diary) to large (insomnia severity) effect sizes for dCBT-i in the adult population.

Despite the growing body of dCBT-i studies conducted in adult populations, RCTs on dCBT-i used in individuals with insomnia in adolescence or early adulthood are rare [28]; thus, determining whether dCBT-i has comparable efficacy to in-person CBT-i in this age group is difficult. In a 2018 systematic review [29], the authors conducted a preliminary analysis with two RCTs. When comparing with a waitlist control group, an effect of medium size on SQ (PSQI, *d* = 0.51) was discovered in one study [30], and another RCT reported promising effects on subjective SQ with small-to-medium effect sizes (SE, SQ, and SOL from a sleep diary), reduced insomnia severity with a large effect size (*d* = 0.98), and improved objective SQ (SE, *d* = 1.09; SOL, *d* = 0.87; total sleep time [TST], *d* = 0.37) as measured through post-treatment actigraphy [31].

These findings emphasize the positive effect of dCBT-i in young people, who may be reluctant to receive psychotherapy and are more engaged with the Internet than other age groups. We collected literature to determine the efficacy of dCBT-i in adolescence and early adulthood. This systematic review and meta-analysis assessed the level of evidence on the effectiveness of dCBT-i in younger age groups, with clinical implications for the management of insomnia in young people.

## 2. Materials and Methods

This study was conducted in accordance with the PRISMA guidelines [32]. The procedure used to identify studies for systematic review and meta-analysis are described herein. The study was preregistered at the International Prospective Register of Systematic Reviews (#CRD42021255923).

### 2.1. Search Strategy

Studies were collected from four key electronic databases—PubMed, Cochrane Library, PsycINFO/Article, and Embase—and were required to meet the following four criteria: (1) the participants were adolescents or active college students; (2) the intervention was digitally delivered cognitive behavioral therapy; (3) the intervention was designed for insomnia or sleep improvement; (4) the applied outcome measurement was standardized. The articles published on PubMed and Cochrane Library between January 1991 and October 2021 were searched, whereas the search duration was set between January 1991 and April 2020 for the studies published on PsycINFO/Article and Embase because of accessibility.

The following search terms were used to identify these criteria: Cognitive Behavioral Therapy [Mesh] or cognitive behavioural therapy or cognitive behavioral therapy or “CBT” or “ICBT” or “CBTi” or “CBT-i” or behav* therap* or be-hav* modification or sleep hygiene or stimulus control or relaxation or sleep restriction AND Adolescent [Mesh] or student or teenager AND “Sleep Initiation and Mainte-nance Disorders” [Mesh] or “insomnia” or “chronic insomnia” or “sleeplessness” or sleep or “sleep initiation” or “sleep maintenance” or “poor sleep” or “sleep problem” or “sleep disturbance” AND digital or mobile or internet* or online or eHealth or mHealth or computerized or computerised or “self-help” AND “Randomized Con-trolled Trial” or “Randomised Controlled Trial” or “RCT” or “Trial” or “Meta-Analysis” or “Meta Analysis” or “Systematic-Review” or “Systematic Review”. 

In addition to forward-searching, we conducted a retrospective search. Bibliographic information from the literature that met the aforementioned criteria was manually searched. Three authors (HJT, JDZ, and YYH) searched the databases and independently examined the articles through their titles or abstracts. Duplicates were removed using an automatic tool (EndNote X9, Clarivate, Philadelphia, PA, USA) by one author (JDZ). After the initial selection, the full-text versions of relevant articles were carefully reviewed and prepared for data extraction. If a disagreement occurred, consensus was obtained from all three reviewers in reference to the inclusion criteria.

### 2.2. Study Selection 

The eligibility criteria for study inclusion were selected in accordance with Population, Intervention, Comparison, Outcomes, and Study (PICOS) guidelines [33] as per the PRIMSA statement. Peer-reviewed studies written in English that met the following criteria were included in the review. **Participants:** The participants were in the age range of youth, including adolescents (10–19 years old; as defined by the World Health Organization; [34]) or currently active college or university students in their early adulthood. Participants reported concerns about their SQ, though this may have not been measured using a standardized SQ questionnaire (e.g., the PSQI or ISI) or may have not met the insomnia disorder diagnostic criteria of the Diagnostic and Statistical Manual of Mental Disorders, Fifth Edition (DSM-V) [2] or International Classification of Sleep Disorders, Third Edition [35]. **Interventions:** The cognitive behavioral therapy applied was designed for SQ or insomnia improvement using multimodal therapies, including cognitive therapy, sleep hygiene, psychotherapy, stimulus control, sleep restriction, and relaxation techniques. The intervention was digitally delivered over the Internet or through a smartphone. The process was fully automated or combined with minimal personal support (e.g., text messages or email reminders). **Comparisons:** The study design for the control group could vary. A waitlist control group of participants must have undergone similar exposure to the intervention, such as the use of self-help material or provision of sleep hygiene education only, for the same treatment duration as the dCBT-i group. **Outcomes:** Sleep-related parameters reported before and after treatment as well as at the follow-up were included. For subjective SQ, standardized sleep questionnaire scores were collected (e.g., PSQI or ISI). Sleep parameters, such as SE or TST, obtained from objective SQ measurements (e.g., actigraphy) were also extracted for analysis. **Study Design:** The study must have had an RCT design. Eligible studies had to have reported the outcomes measured before and after treatment as well as at the follow-up, with the outcomes reported in means and standard deviations (or standard errors) for experimental and control groups. Three authors independently extracted the data and crosschecked the results. Discussions were held and consensuses reached to address any disagreement.

### 2.3. Analytical Strategy

The available results that measured SQ and insomnia severity using standardized questionnaires after treatment or at the follow-up were pooled to identify the short- and long-term effects of dCBT-i on subjective SQ (the primary outcome); the objective SQ findings SE and TST, recorded using actigraphy or polysomnography, were computed to determine the efficacy of dCBT-i in regard to sleep structure (the secondary outcome). For subsequent data synthesis, TST was set in minutes, with results reported in hours being automatically converted into the desired unit. 

The sensitivity analysis was conducted on SQ when a possible confounding factor was presented on the eligible studies (e.g., participant’s characteristics); moreover, we further differentiated the enrolled populations in the eligible studies into the following subgroups for analysis and to identify the differential effects of dCBT-i on different insomnia populations: (1) participants who met the criteria of insomnia disorder (either through clinical diagnosis or a standardized insomnia questionnaire; primary insomnia), and (2) participants who reported having other major issues alongside concern about their SQ (i.e., comorbid insomnia). The meta-analysis was performed when results were available for at least two studies.

### 2.4. Quality Assessment

Heterogeneity assessment, effect size calculation, and publication bias were tested using Review Manager version 5.4(Cochrane Collaboration, Copenhagen, Denmark). Higgins and Thompson’s I^2^ was calculated to test between-study heterogeneity [36]. The I^2^ was used to assess the variance in the effect size on the basis of Cochran’s Q-statistic. An I^2^ of 0% to 40%, 41% to 74%, or 75% to 100% represented low, medium, or considerable heterogeneity, respectively [37]. A random-effect model was implemented for outcome assessment.

The use of standardized mean differences to calculate the pooled effect size minimized the bias produced by the small sample, and Hedges’s *g* [38,39] and a 95% confidence interval (CI) were estimated. The mean and standard deviation in the experimental and control groups after treatment or at the follow-up were utilized for effect size calculations. The pooled effect size was then computed using inverse-variance weighting to increase the precision. For subjective SQ measurement, a negative effect size indicated an effect in the experimental group, whereas a positive effect size for objective SQ indicators (SE or TST) represented an improvement in sleep structure in the treatment group. When Hedges’s *g* for the effect size ranged from 0 to 0.32, 0.33 to 0.5, or 0.56 to 1.2, a small, moderate, or large effect was indicated, respectively [40].

Moreover, to detect the treatment effect and to estimate the clinical significance [41], the number needed to treat (NNT) was computed using the Kraemer and Kupfer method [42] for subjective SQ. In brief, the effect size was transformed into the probable number of patients who would benefit from this treatment in comparison with those treated in the traditional manner. A smaller NNT represents greater effectiveness of a particular intervention or treatment. For instance, an NNT of 3 indicates that one out of three patients would respond to the treatment. However, if the NNT is negative, the treatment or intervention had a negative effect; for example, an NNT of −10 indicates that a negative treatment effect would occur in one out of 10 patients. The NNT calculation was conducted using R (version 4.1).

To determine whether the conclusion derived from a meta-analysis was reliable, we evaluated the methodological quality of the studies. The risk of bias—including selection (random sequence generation and allocation concealment), performance (blinding the participants and personnel), detection (blinding of the outcome data), attrition (incomplete outcome data), and reporting biases (selective reporting)—was independently assessed by two of the authors (HJT and JDZ). If a consensus could not be reached, a final decision would be reached following a discussion with the author (TFH). The level of bias risk was classified as low, high, or unclear bias.

## 3. Results

**Figure 1:**
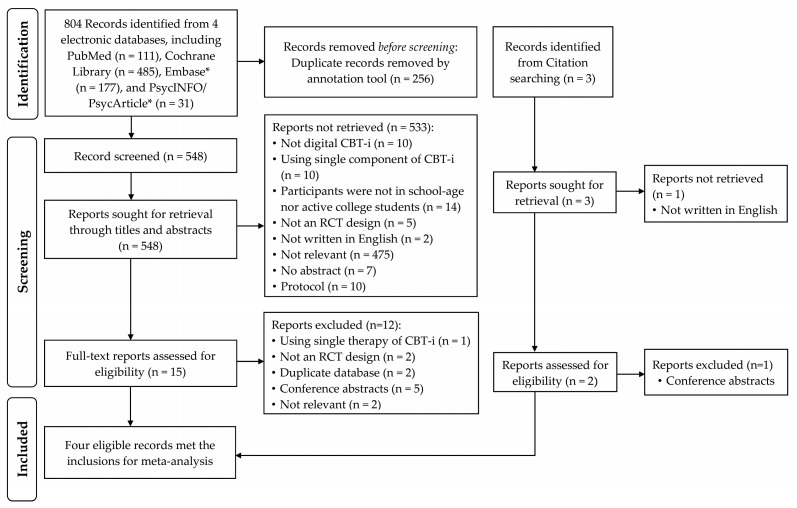
PRISMA study inclusion flowchart. Studies published between January 1991 to October 2021 were collected from four key electronic databases. CBT-i, cognitive behavioral therapy for insomnia; RCT, randomized controlled trial. *, Search duration was set from January 1991 to April 2020.

A total of 804 records from the electronic databases were initially included for title and abstract inspection after 256 duplicates had been removed, and 16 studies underwent full-text screening. A total of four RCTs [30,43,44,45] met the inclusion criteria and were included in this review (Figure 1). 

### 3.1. Quality Assessments

Visual inspection of the funnel plot indicated low publication bias in the current meta-analysis. Study appraisal was conducted for six domains, including participant blinding and selection, detection, attrition, and reporting biases. The raters (HJT, JDZ, TFH) agreed that the studies targeted in this review had generally low risk of bias.

Three out of four studies (75%) included power calculations [30,43,45], and all studies applied intention to treat in data analyses. As listed in Table 1, 75% of the studies provided sufficient description of the key elements of their study design for review and employed adequate procedures for randomization, allocation concealment, participant blinding, outcome assessment, and selective reporting. One study (25%), however, did not conduct allocation concealment or participant blinding [30] and reported a single indicator of sleep outcome measurement, resulting in potential risk of selective-reporting bias (no protocol registration; unclear bias).

### 3.2. Study Characteristics

A total of 3970 participants were recruited into the studies, with 1999 participants allocated into a dCBT-i group (mean age = 15.3–24.8 years; 1433 [71.69%] were women, and the sex of 17 participants was unidentified) and 1971 into a control group (mean age = 15.9–24.6 years; 1386 [70.32%] were women, and the sex of 19 participants was unidentified). Three studies (75%) applied a treatment program with a duration of six weeks [30,43,45], and the other study used a four-week intervention [44]. Three studies (75%) conducted follow-up evaluations at 2 [45], 3 [43,44], 6 [45], and 12 months [45] after treatment completion. All studies applied a standardized sleep questionnaire (e.g., PSQI or ISI) for subjective SQ evaluation as the primary outcome measurement, and two studies also recorded that participants’ objective sleep patterns were measured using a wrist-worn actigraph before and after treatment [44,45].

The four included studies are detailed in Table 2. Descriptions of the studies are ordered herein on the basis of their year of publication. 

(1) The first study was conducted by Morris et al. (2015) [30]. This study recruited undergraduates from the University of Bristol in the United Kingdom who were experiencing stress and expressed a desire to learn coping skills for stress management. Evidence requirements for a determination of insomnia or poor SQ are not listed in the participant inclusion criteria (i.e., insomnia diagnosis or use of standardized sleep questionnaires). Participants were randomly assigned into three groups, namely waitlist control, “anxiety-relief,” and “insomnia-relief” groups.

The “insomnia-relief” group received a commercial dCBT-i program featuring automatic and Internet-based self-help materials. This program consisted of seven modules with components of CBT-i, including psychoeducation, sleep hygiene, stimulus control, sleep restriction, relaxation techniques, and cognitive imagery. Participants completed each module, in order, within six weeks and received weekly text and email reminders. The PSQI was used before and after treatment for sleep outcome measurement. Participants were aged in their early 20s, with 47 (70% women) and 48 (60% women) participants in the control and dCBT-i groups, respectively. Among them, 6.38% of control participants (*n* = 3) did not complete the posttreatment evaluation. In total, 14.58% (*n* = 7) and 10.42% (*n* = 5) of the dCBT-i group were withdrawn from the analysis due to no posttreatment evaluation and not following the dCBT-i instructions, respectively.

(2) The second study was published by Freeman et al. (2017) [43]. This study recruited students from 26 universities in the United Kingdom and screened them using a standardized sleep questionnaire (the Sleep Condition Indicator, SCI) [46]. This questionnaire was developed by one of their authors and based on the diagnostic criteria of insomnia disorder in the DSM-V. Participants older than 18 years who had an SCI score less than or equal to 16 were randomly assigned to an experimental or waitlist control group. The Sleepio dCBT-i program was used in this study; it includes all key components of CBT-i, including sleep restriction, stimulus control, relaxation, cognitive techniques (paradoxical intentions, belief restructuring, mindfulness, and putting the day to rest), and sleep education (mechanisms of sleep and sleep education). In the web-based dCBT-I, participants complete their daily sleep diary and access certain self-help materials using their smartphone, including relaxation audio. Moreover, the dCBT-i program features an animated therapist that participants can interact with and that provides personalized sleep schedule suggestions. An email or text message is sent to the user if they do not attend a session to promote engagement with the program.

The SCI-8 (SCI with eight items; primary outcome), ISI (secondary outcome), and the Disturbing Dreams and Nightmare Severity Index [47] were employed to quantify the sleep-related treatment outcomes at week 0 (pretreatment), week 10 (posttreatment), and week 22 (follow-up). Participants were aged in their mid-20s, with 1864 (71% women) and 1891 participants (72% women) in the control and dCBT-i groups, respectively. The dropout rate was high overall, though higher in the dCBT-i group than in the control group. At the posttreatment stage, only 1142 (61.27%) and 733 participants (38.76%) in the control and dCBT-i group, respectively, reported for primary outcome evaluation. Additionally, only 971 (52.09%) and 603 participants (31.89%) in the control and dCBT-i group, respectively, completed their primary outcome evaluation at the follow-up (week 22). Moreover, 31.15% of the participants (*n* = 589) in the dCBT-i group completed zero sessions, and only 17.50% of the participants (*n* = 331) finished the course.

(3) The third study was performed by Fucito et al. (2017) [44]. Study participants were undergraduate students who reported problems with alcohol consumption and concerns about SQ (yes or no); no insomnia diagnosis or use of a standardized sleep questionnaire was required for inclusion. Participants were randomly assigned to a control or dCBT-i group. The dCBT-i program used in the study, named Call it a Night, was developed by the authors and focused on the management of alcohol consumption and providing a sleep intervention. This web-based program comprised four modules delivered weekly covering behavioral, cognitive, and educational components, including sleep hygiene, stimulus control, cognitive techniques, relaxation, and psychoeducation. Participants received personalized sleep schedule suggestions, and a daily email reminder was sent to promote program engagement. The control group also participated in a four-week intervention (“Healthy Behaviors”) with basic health-promotion recommendations and sleep education to maintain participant engagement. This program controlled for the treatment duration and number of modules to match the dCBT-i group, but it employed simplified content without personalized feedback.

The PSQI and Patient-Reported Outcomes Measurement Information System Sleep-Related Impairment Short-Form [48] evaluations for SQ and sleep-related impairment were completed by the participants at week 0 (pretreatment), week 5 (immediately posttreatment), and week 12 (follow-up). The treatment outcome of objective SQ, measured using actigraphy, was also reported. The participants were asked to wear a wrist actigraph at week zero (pretreatment) and week four (the last week of treatment) to record their sleep patterns, with event markers used to identify the main sleep phase. Participants were in their early 20s, with 21 participants (48% women in both groups) in both the control and dCBT-i groups, respectively. In both groups, two participants did not complete the follow-up evaluation, resulting in a dropout rate of 9.52%. Moderate adherence was reported in both groups, with only 19.04% and 23.80% of the control and dCBT-i participants failing to complete at least 80% of the course, respectively. The authors observed no differences in subjective or objective sleep characteristics between those who completed the treatment and those who did not. However, participants who completed the full course were more likely to be women and consume less alcohol.

(4) The most recent RCT study was published in 2018 by De Bruin and colleagues [45]. Study participants were from a community population and were in middle school or later grades. All eligible participants were adolescents (12–19 years old) who met the DSM-V criteria for insomnia disorder and were not on medication. They were randomly assigned to three groups, namely waitlist control, in-person CBT-i therapy, and dCBT-I groups.

The dCBT-i program comprised six sessions on psychoeducation, sleep hygiene, sleep restriction, stimulus control, cognitive therapy, and relaxation. Participants were required to visit the web-based program weekly and revisit all content as part of a follow-up at two months for a booster effect. Participants completed a session with a sleep therapist following the second week of the intervention, and the program provided personalized sleep schedules and feedback. A sleep diary and the Holland Sleep Disorder Questionnaire–Insomnia Subscale [49] were the primary sleep outcomes for subjective SQ evaluation, with objective measurement achieved using a wrist-worn actigraph that recorded the sleep pattern of each participant at baseline and after treatment. Participants in the dCBT-i group were asked to complete the subjective and objective SQ assessment at baseline, after treatment, and at the follow-ups (2, 6, and 12 months after treatment). The SQ of the control participants was measured at baseline, after treatment, and at a follow-up (two months after treatment).

The control and dCBT-i groups consisted of 39 (72% women) and 39 participants (85% women), respectively. The dropout rate was low (<5%), with 100% (*n* = 39) and 97.44% (*n* = 38) of the control and dCBT-i participants completing the posttreatment evaluation, respectively. Additionally, 94.87% (*n* = 37) and 97.44% (*n* = 38) of the control and dCBT-i participants completed the follow-up measurement at two months, respectively. No significant differences in age, sex, or insomnia symptoms were observed between the intervention and nonintervention participants.

### 3.3. Short-Term and Long-Term Effects of dCBT-i 

The self-reported and actigraphy-measured SQ reported in the studies enabled us to test the efficacy of dCBT-i in regard to subjective and objective SQ in young people. To identify the short- and long-term effects of dCBT-i among young individuals with insomnia, the pooled effect sizes after the treatment and at the follow-up were calculated. Three studies conducted follow-up evaluations, specifically at week 22 from treatment initiation (equivalent to three months after the posttreatment evaluation) [43] and at three months after treatment completion [44]. One study performed follow-ups at 2, 6, and 12 months after treatment [45]; the results of the two-month follow-up were included to match the time points of the other studies and for calculation of the pooled effect size for long-term effects. The follow-up evaluation focused on subjective SQ because no study reported objective SQ assessment at the follow-up stage.

**Figure 2:**
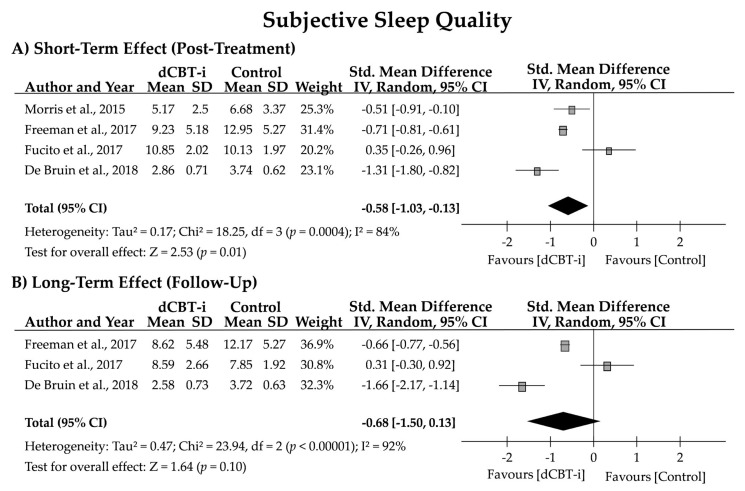
Forest plot for the short- and long-term effects of dCBT-i on subjective sleep quality (SQ). Subjective SQ was evaluated using the Pittsburgh Sleep Quality Index (PSQI; Morris et al., 2015 [30]; Fucito et al., 2017 [44]), Insomnia Severity Index (ISI; Freeman et al., 2017 [43]), or Holland Sleep Disorder Questionnaire-Insomnia (HSDQi; De Bruin et al., 2018 [45]). SD, standard deviation; CI, confidence interval.

The studies evaluated their participants’ subjective SQ through sleep diaries and various sleep questionnaires. Scores on the PSQI and ISI after treatment or at the follow-up evaluation were employed as the primary sleep outcomes for meta-analysis. The effect size for subjective SQ was significant for the posttreatment evaluation (*n* = 4; *g* = −0.58 [95% CI = −1.03, −0.13]; z = 2.53, *p* = 0.01) but not for the follow-up evaluation (*n* = 3; *g* = −0.68 [95% CI = −1.50, 0.13]; z = 1.64, *p* = 0.10). High heterogeneity in the subjective SQ after treatment (84% and 92%) and at the follow-up was also noted (Figure 2).

In terms of clinical significance, the NNTs of post-treatment subjective SQ were 3.55, 2.60, −5.12, and 1.55 in the four studies, with a pooled NNT of 0.65. Likewise, the NNTs of follow-up subjective SQ were 2.78, −5.76, and 1.32, with a pooled NNT of −0.33. After exclusion of the negative value of the NNT, the pooled NNTs were 2.57 and 2.05 for posttreatment and follow-up, respectively.

**Figure 3:**
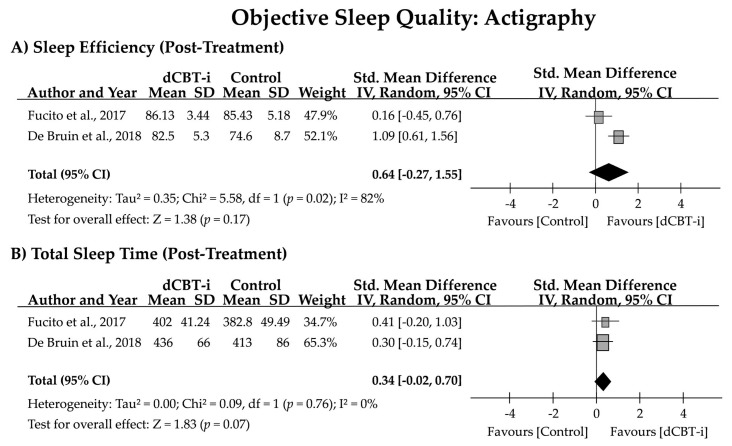
Forest plot of effect size for the effect of dCBT-i on objective SQ measured using actigraphy. SD, standard deviation; CI, confidence interval.

The two studies that reported sleep parameters measured using actigraphy at the post-treatment assessment enabled examination of the effect of dCBT-i on objective SQ (Figure 3). Heterogeneity estimates revealed an I^2^ of 82% and 0% for SE and TST, respectively. No statistical significance was observed for SE (*g* = 0.64 [95% CI = −0.27, 1.55]; z = 1.38, *p* = 0.17) or TST (*g* = 0.34 [95% CI = −0.02, 0.70]; z = 1.83, *p* = 0.07) after treatment.

### 3.4. Effects of dCBT-i on Insomnia Subtypes

To test whether the efficacy of dCBT-i differed between insomnia subtypes, the pooled effect sizes of the effects of dCBT-i on these subtypes were analyzed. Subjective SQ was the primary outcome in this subgroup analysis. Two studies applied insomnia determination criteria for participant recruitment, indicating that participants with an SCI less than or equal to 16, or who met the DSM-V definition of insomnia disorder, were included. Hence, the participants from these studies were categorized as individuals with primary insomnia. Participants from the other two studies reported problems in addition to sleep disturbance, such as stress management or heavy alcohol consumption. Thus, the participants from these studies were categorized as individuals with comorbid insomnia. 

**Figure 4:**
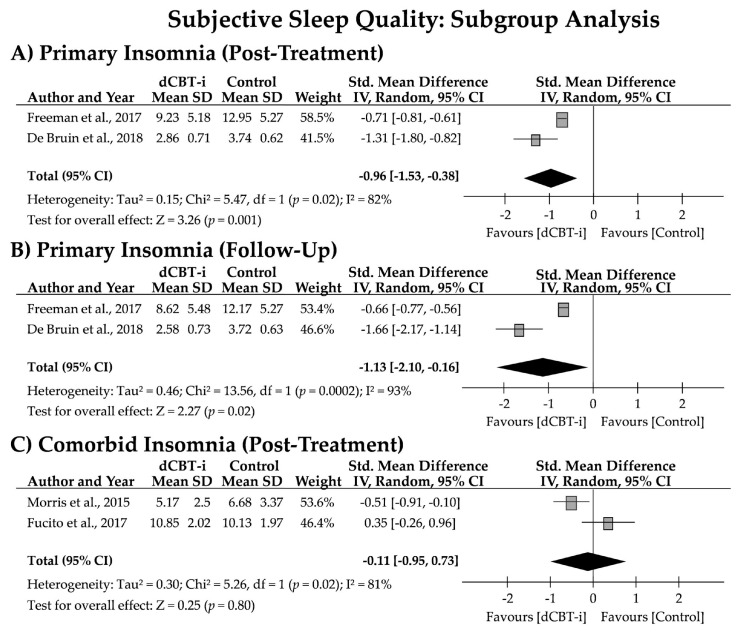
Forest plot for short- and long-term effects of dCBT-i on subjective sleep quality among insomnia subtypes. Subjective sleep quality was evaluated using the PSQI (Morris et al., 2015 [30]; Fucito et al., 2017 [44]), ISI (Freeman et al., 2017 [43]), or HSDQi (De Bruin et al., 2018 [45]). SD, standard deviation; CI, confidence interval.

As depicted in Figure 4, high heterogeneity was observed in subjective SQ (81–93%) in the subgroup analysis. The pooled effect sizes for primary insomnia after treatment (*g* = −0.96 [95% CI = −1.53, −0.38]; z = 3.26, *p* = 0.001) and at the follow-up (*g* = −1.13 [95% CI = −2.10, −0.16]; z = 2.27, *p* = 0.02) were significant, but the effect size for comorbid insomnia after treatment failed to reach significance (*g* = −0.11 [95% CI = −0.95, 0.73]; z = 0.25, *p* = 0.80). Moreover, a pooled NNT of 2.08 (2.60 and 1.55) and 2.05 (2.78 and 1.32) was noted for primary insomnia after treatment and at the follow-up, respectively. Two studies on comorbid insomnia, however, obtained contrasting results, with NNTs of 3.55 and −5.12 at the post-treatment assessment. 

### 3.5. Sensitivity Analysis

Although four studies were identified in accordance with the PICOS, one study conducted by Fucito et al., 2017 (i.e., the third study described in Section 3.2 Study Characteristics) might display a confounding factor in terms of the participant’s characteristics. Despite that this study employed a dCBT-i program to intervene with sleep problems among university students who reported having drinking issues and were concerned about sleep quality, the participant’s characteristics of this particular study would be different from other studies. A sensitivity analysis was then conducted to identify the treatment effect on SQ. 

**Figure 5:**
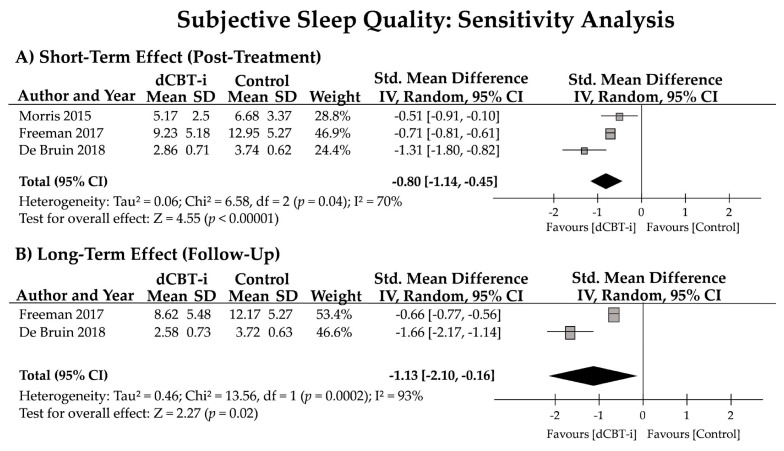
Sensitivity analysis of the short- and long-term effects of dCBT-i on subjective sleep quality (SQ). Subjective SQ was evaluated using the Pittsburgh Sleep Quality Index (PSQI; Morris et al., 2015 [30]), Insomnia Severity Index (ISI; Freeman et al., 2017 [43]), or Holland Sleep Disorder Questionnaire-Insomnia (HSDQi; De Bruin et al., 2018 [45]). SD, standard deviation; CI, confidence interval.

After removing the study conducted by Fucito et al., 2017 and pooling the other three studies, the effect size for subjective SQ was significant for the post-treatment evaluation (*n* = 3; *g* = −0.80 [95% CI = −1.14, −0.45]; z = 4.55, *p* < 0.01) and for the follow-up evaluation (*n* = 2; *g* = −1.13 [95% CI = −2.10, −0.16]; z = 2.27, *p* = 0.02). High heterogeneity in the subjective SQ after treatment and at the follow-up was also noted (70% and 93%; Figure 5).

## 4. Discussion

This meta-analysis assessed the efficacy of digitally delivered, multicomponent CBT-i in young individuals with insomnia. The crucial nature of this topic is not reflected in the limited RCTs that have been conducted (*n* = 4, as of October, 2021). Three of the four studies were judged to have low risk of bias in all six domains following study appraisal, and one study was judged to have high risk of bias related to allocation concealment and blinding. The key findings of this analysis are as follows. (1) dCBT-i exhibited a medium positive effect (*n* = 4; *g* = −0.58, *p* = 0.01) on subjective SQ immediately after treatment when comparing the intervention and control groups. Such a treatment effect was enlarged when sensitivity analysis was applied (*n* = 3; *g* = −0.80, *p* < 0.001). (2) No significant treatment effect of dCBT-i at the follow-up was discovered (*n* = 4; *g* = −0.68, *p* = 0.10), but it showed a significant long-term treatment effect after employing the sensitivity test (*n* = 3; *g* = −1.13, *p* = 0.02). (3) No significant improvements in objective SQ (i.e., SE or TST) were noted. (4) In participants with clinically significant insomnia, the effect size was larger in regard to short-term (*g* = −0.96, *p* = 0.001) and long-term efficacy (*g* = −1.13, *p* = 0.02) compared with the control intervention. (5) Among individuals with comorbid insomnia, dCBT-i had no significant positive effect on subjective SQ (*g* = −0.11, *p* = 0.80).

Despite the medium-to-large effect sizes for subjective SQ and indication of significant improvement in SQ, the scores for subjective SQ, evaluated using the PSQI after treatment or at the follow-up, remained within the range of poor SQ (PSQI ≥ 5), as reported in two of the studies [30,44]; however, significant improvement in insomnia severity (i.e., scores below those determining clinical significance) was reported by Freeman et al. [43] and De Bruin et al. [45]. These findings suggest that dCBT-i has a positive effect on the management of insomnia severity but may be less effective in altering general SQ. Regarding clinical significance, the pooled NNT of 2.57 (range: 1.55–3.55) computed from three studies indicated that one out of three patients would benefit from dCBT-i treatment, whereas one study indicated the opposite (NNT = −5.12). This particular study also downgrades the effect sizes for short-term and long-term efficacy of dCBT-i on subjective SQ, as it was evident by sensitivity analysis. This one study applied dCBT-i to university students who reported heavy alcohol consumption and sleep problems [44], which suggests that the motivation for sleep improvement is crucial for successful treatment outcomes [25], particularly for digital psychobehavioral interventions without therapist supervision. 

In contrast to the success in enhancement of subjective SQ, no significant treatment effect was detected on objective SQ, measured through actigraphy in a posttreatment assessment. Although the meta-analysis was performed with data from two studies and must be interpreted cautiously, no significant improvement in either SE (*g* = 0.64 [95% CI = −0.27, 1.55], *p* = 0.17) or TST (*g* = 0.34 [95% CI = −0.02, 0.70], *p* = 0.07) was detected following dCBT-i treatment. However, a trend of increasing TST with dCBT-i was noted because the 95% CI had a narrow margin to the null hypothesis value. In fact, no significant treatment effect on objective sleep parameters was expected. This finding could have resulted from the paucity of randomized controlled studies in this population; however, similar findings were reported in a meta-analysis that examined the effect of in-person CBT-i on objective sleep parameters in adults [50]. The authors determined robust improvement in self-reported sleep structures from sleep diary data but not from objective measurement. No such effects on sleep parameters were observed when using polysomnography (five RCTs were included). For sleep parameters recorded through actigraphy, a small effect size was recorded for SOL (*g* = −0.28), but a significant negative effect on TST (*g* = −0.51; i.e., reduced TST after treatment) was also detected.

However, these findings draw attention to the “treatment effect”. Insomnia with objective short sleep duration is the most severe insomnia subtype [51] and is associated with increased risks of cardiovascular incidents, diabetes, and mortality rates compared with insomnia with adequate sleep duration. The lack of evidence to support significant improvement in objective sleep structure through CBT-I leads to difficulty in identifying whether the intervention has a protective effect in terms of their health condition and reduced risks of cardiovascular disease, mortality, and dementia [51,52,53,54]. Further longitudinal studies are required to explore the protective effect of face-to-face CBT-i or dCBT-i on the health of patients with insomnia.

As a consequence of the self-reported criteria for insomnia diagnosis [2] and the aims of rebuilding the perception of sleep and shaping healthy sleep behaviors being at the core of CBT-i [16], this therapy focuses on minimizing the discrepancy between subjective and objective SQ [55], as well as increasing sleep drive; however, little has been conducted in terms of rebuilding objective sleep structure. Moreover, changes in sleep architecture can be viewed as a final product of the modification of sleep-related microstructures. One study investigated the changes in sleep patterns and sleep-related cortical activities before and after in-person CBT-i treatment in patients with insomnia. No significant improvement of polysomnography-measured sleep structures was reported in regards to the increase of deep sleep (stage 3) and reduction of shallow sleep (stage 1) among patients [56,57]. However, significant changes in electroencephalogram during non-rapid-eye-movement (NREM) sleep were observed [56,57,58], suggesting that CBT-i altered the microstructure but not the macrostructure of sleep.

The most notable finding was the efficacy of dCBT-i in insomnia subtypes. Although dCBT-i had a medium effect (*g* = −0.58) on subjective SQ (measured after treatment), this efficacy may be more attributable to the individuals with primary insomnia than those with comorbid insomnia. In the subgroup analysis, we differentiated participants with insomnia diagnosis without comorbidity or co-occurring issues (primary insomnia) and those experiencing major problems other than insomnia (i.e., stress or heavy alcohol consumption; comorbid insomnia). The effect size was large for participants with primary insomnia (*g* = −0.96) immediately after dCBT-i treatment. Moreover, the dCBT-i treatment effects persisted after two to three months (*g* = −1.13), demonstrating the significant short- and long-term treatment efficacy of dCBT-i in young primary insomniacs. Conversely, no such treatment effect was discovered in individuals with comorbid insomnia (*g* = −0.11); no results were available to compute the long-term effects for those with comorbid insomnia. It should also be noted that because only two studies were available for each subgroup, resulting in a high heterogeneity of analysis (I^2^ > 75%), more controlled studies in investigations of dCBT-I among insomnia subtypes are strongly encouraged. Nevertheless, these preliminary findings support those stating that dCBT-i is less effective and has higher attrition among participants with sleep disorders, low insomnia severity, and psychiatric comorbidities [25].

The questionnaires used to evaluate subjective SQ for the results of the subgroup analysis must also be reviewed. Notably, to pool the effect size of subjective SQ in the primary insomnia group, ISI [43] or HSDQi scores were employed [45], but the two studies in the comorbid insomnia group reported PSQI scores for their primary sleep outcome [30,44]. The ISI and HSDQi are designed to measure the severity of insomnia and are less strongly affected by other factors (e.g., an individual’s beliefs about sleep) [59]. The PSQI has been widely used to measure individuals’ general SQ over a month-long period. A score greater than or equal to 5 on the PSQI indicates poor SQ [4]. However, the PSQI reflects general SQ and sleep-related distress but not insomnia [60], and patients with insomnia and psychiatric comorbidity tend to report poorer SQ than primary insomniacs as a result of significant negative recall bias [61]. Not only does recall bias affect these results, but sleep beliefs can also influence recall. The cognitive therapy within CBT-i aims to alter unrealistic sleep beliefs and reshape sleep perception, which—in view of how raters interpret their sleep—can be confounding factors for the PSQI [59]. Hence, the PSQI may not be an appropriate indicator of insomnia severity [60] nor a reliable outcome measurement for CBT-i [59].

Although the application of dCBT-i in the management of insomnia in young people is promising, caution must be exercised when interpreting and generalizing the present findings. Only four RCTs were available for review, and a significant high heterogeneity (I^2^ > 75%) was presented in every outcome assessment. The meta-analysis on TST and SE in relation to objective SQ was only performed with two studies, which limits the robustness of the findings. A similar issue occurred when conducting subgroup analysis. More in-depth RCTs are required to update and reexamine these findings. Furthermore, most of the studies applied retrospective outcome measurement (i.e., recalled SQ during a period of time in the past), with only one study providing prospective outcomes measured using sleep diaries. Consequently, recall bias was inevitable. The use of prospective and retrospective outcome measurements to evaluate day-to-day sleep dynamics and overall improvement is encouraged.

Additionally, three of the four studies focused on college students, and only one study examined the treatment effects on adolescents. Although different sleep patterns could be displayed between adolescents or young adults, we only included studies with active college students to minimize the potential confounding factors caused by lifestyle; moreover, the forest plot in Figure 3 showed that the study group with adolescents (i.e., De Bruin et al., 2018) presented a similar treatment effect of the study performed on college students, with both of the studies using the Insomnia Severity Index to measure treatment effects (i.e., Freeman et al., 2017). Thus, we did not exclude this study from our meta-analysis. Lastly, a lack of information on the medication status of participants (only one study provided this, stating that participants were medication-free) resulted in difficulty determining how dCBT-i interplays with sleep medication use in young people with insomnia; whether dCBT-i plays a role in medication tapering remains unclear. 

As we systematically searched the literature that reported the effects of dCBT-i in youth, including adolescents or college students, the paucity of the RCTs in this population was unexpected. Through an analysis of the effect of dCBT-i on different dimensions—including insomnia subtypes, subjective SQ, and objective findings—we highlight the necessity of increasing sleep-health program accessibility in this population and strongly recommend more quality studies to update and examine the subjective and objective improvements that can be made by digital medicine. Moreover, to achieve personalized digital sleep medicine [62] or digital phenotyping insomnia disorder [63,64], the future development for the dCBT-i with advanced technologies is recommended, including digital devices (e.g., smartphones, wearables, or other connected sensors) [65] and artificial intelligence (e.g., digital sleep questionnaire) [66,67,68]. The former passively collects the data from the user and allows the latter to proactively identify the compliance and to generate a personalized treatment plan [69] and outcomes or a relapse prediction [70]. Such a framework has been demonstrated by a recent study [71] and showed promising utility in the improvement of patient motivation, discipline, and clinical performance.

Overall, our findings support the acceptability of digital psychobehavioral therapy for those of a young age and with high personal willpower to persist with treatment [25]. Digital medicine is still developing, and the nature of dCBT-i (highly structured content, less flexibility, and no therapist supervision) may not be an appropriate intervention for patients with complex insomnia symptoms that require more professional assistance [23]. For instance, a patient with insomnia and evident suicidal intentions or comorbid psychiatric disorder must be treated with face-to-face CBT-i. In addition, dCBT-i is not a replacement for in-person CBT-i, but it is a complementary intervention or initial step into mental health treatment indicated by the stepped care model [72]. With the advantage of accessibility, dCBT-i can expose people with sleep problems to treatment content in real time and then be followed by therapy delivered by trained therapists or sleep specialists if necessary. 

## 5. Conclusions

This study reviewed the efficacy of dCBT-i in young people. These preliminary findings showed that dCBT-i has a medium-to-large effect in the short- and long-term subjective SQ improvement among young insomniacs. Contrarily, the intervention exhibited small treatment effects on objective SQ. Although few studies were available for review, the literature continues to grow. The current study presented the preliminary findings on examining the effectiveness of digital sleep medicine for the younger generation, highlighting the paucity of the RCTs performed in this population, and it recommended the future development for dCBT-i to interact with current trending technologies for user engagement and outcome prediction. 

## Figures and Tables

**Table 1:** Risk of bias summary on each risk of bias item for each included study.

	Random Sequence Generation (Selection Bias)	Allocation Concealment (Selection Bias)	Blinding of Participants	Blinding of Outcome Assessment (Detection Bias)	Incomplete Outcome Data (Attrition Bias)	Selective Reporting (Reporting Bias)
Morris et al., 2015 [30]	+	+	+	+	+	?
Freeman et all., 2017 [43]	+	+	+	+	+	+
Fucito et al., 2017 [44]	+	+	+	+	+	+
De Bruin et al., 2018 [45]	+	+	+	+	+	+

(+), (−), and (?) indicate that the study presented low, high, or unclear bias in their applied method, respectively.

**Table 2:** Randomized controlled trials of digital cognitive behavioral therapy for insomnia enrolled for meta-analysis.

Author (Year) Location	Sample Size (F%) Mean Age (SD)	Recruitment Population	Group Allocation: Completed at Posttreatment (Dropout N.)	Therapy Delivery	Components of dCBT-i	Sleep-Related Outcomes
Morris et al. (2015) [30] UK	dCBT-i: *N* = 48 (60%) 20.69 ± 2.61 y/o WC: *N* = 47 (70%) 20.27 ± 1.56 y/o	Undergraduate students at the University of Bristol who want to learn stress management	dCBT-i: 36 (12) WC: 44 (3)	“Insomnia-relief” 7 modules; 6 weeks; text- and email-reminder sent weekly	SH, SC, SR, RT, PE	PSQI *
Freeman et al. (2017) [43] UK	dCBT-i: *N* = 1891 (72%) 24.8 ± 7.7 y/o WC: *N* = 1864 (71%) 24.6 ± 7.6 y/o	University students from 26 universities in the UK who were with insomnia; SCI ≤ 16	dCBT-i: 733 (1158) WC: 1142 (722)	“Sleepio” 6 sessions, on average, of 20 min; weekly; web-based or via smartphone; email- or text-reminder sent to delayed responders	SH, SC, SR, RT, PE, CT	ISI * SCI-8 DDNSI
Fucito et al. (2017) [44] USA	dCBT-i: *N* = 21 (48%) 20.71 ± 1.42 y/o HC: *N* = 21 (48%) 20.33 ± 1.2 y/o	Undergraduate students with heavy-drinking and concern about sleep	dCBT-i: 19 (2) HC: 19 (2)	“Call it a Night” 4 modules; weekly; web-based; email reminder sent daily	SH, SC, RT, PE, CT	PSQI * PROMIS-SRI-SF Actigraphy *
De Bruin et al. (2018) [45] The Netherlands	dCBT-i: *N* = 39 (85%) 15.3 ± 1.4 y/o WC: *N* = 39 (72%) 15.9 ± 1.6 y/o	12–19 y/o adolescents who met DSM-V insomnia criteria; medication-free	dCBT-i: 38 (1) WC: 39 (0)	dCBT-i 6 weeks; 90 min per session; web-based; 15 min online chat with therapist; text reminder sent to delayed responders	SH, SC, SR, RT, PE, CT	Sleep Diary HSDQi * Actigraphy *

Note. dCBT-i, digital cognitive behavioral therapy for insomnia; F%, percentage of female participants in the group; y/o, years old; WC, waitlist control group; HC, sleep-hygiene control group; DSM-V, Diagnostic and Statistical Manual of Mental Disorders, Fifth Edition; SH, sleep hygiene; SC, stimulus control; SR, sleep restriction; RT, relaxation techniques; CT, cognitive technique; PE, psychoeducation; PSQI, Pittsburgh Sleep Quality Index; SCI, Sleep Condition Indicator; ISI, Insomnia Severity Index; DDNSI, Disturbing Dreams and Nightmares Severity Index; PROMIS-SRI-SF, Patient-Reported Outcomes Measurement Information System Sleep-Related Impairment Short-Form; HSDQi, Holland Sleep Disorder Questionnaire-Insomnia symptoms. * Results used for data synthesis in the meta-analysis.

## Data Availability

Not applicable.
